# The EHA Research Roadmap: Hematopoietic Stem Cells and Allotransplantation

**DOI:** 10.1097/HS9.0000000000000714

**Published:** 2022-04-29

**Authors:** Willem Fibbe, Rosa Bernardi, Pierre Charbord, Daniela Krause, Cristina Lo Celso, Simón Méndez-Ferrer, Christine Mummery, Robert Oostendorp, Marc Raaijmakers, Gerard Socié, Frank Staal, Andrea Bacigalupo

**Affiliations:** 1 Department of Internal Medicine and Nephrology, Leiden University Medical Center. Leiden, the Netherlands; 2 IRCCS San Raffaele Scientific Institute, Milan, Italy; 3 Sorbonne Université, Paris, France; 4 Goethe University Frankfurt and Georg-Speyer-Haus, Frankfurt am Main, Germany; 5 Department of Life Sciences and Centre for Haematology, Imperial College London, United Kingdom; 6 University of Cambridge and NHSBT, Cambridge, United Kingdom; 7 Department of Anatomy & Embryology, Leiden University Medical Center, Leiden, the Netherlands; 8 Department of Internal Medicine III, Technical University of Munich, School of Medicine, Munich, Germany; 9 Erasmus MC, Rotterdam, the Netherlands; 10 Hospital Saint Louis, APHP & University of Paris, France; 11 Department of Immunology, Leiden University Medical Center, Leiden, the Netherlands; 12 Fondazione Universitaria Policlinico Gemelli, Rome, Italy

*In 2016, the European Hematology Association (EHA) published the EHA Roadmap for European Hematology Research*^1^ *aiming to highlight achievements in the diagnostics and treatment of blood disorders, and to better inform European policy makers and other stakeholders about the urgent clinical and scientific needs and priorities in the field of hematology. Each section was coordinated by one to two section editors who were leading international experts in the field. In the five years that have followed, advances in the field of hematology have been plentiful. As such, EHA is pleased to present an updated Research Roadmap, now including eleven sections, each of which will be published separately. The updated EHA Research Roadmap identifies the most urgent priorities in hematology research and clinical science, therefore supporting a more informed, focused, and ideally a more funded future for European hematology research. The eleven EHA Research Roadmap sections include Normal Hematopoiesis; Malignant Lymphoid Diseases; Malignant Myeloid Diseases; Anemias and Related Diseases; Platelet Disorders; Blood Coagulation and Hemostatic Disorders; Transfusion Medicine; Infections in Hematology; Hematopoietic Stem Cell Transplantation; CAR-T and Other Cell-based Immune Therapies; and Gene Therapy.*

## ALLOGENEIC STEM CELL TRANSPLANTATION

### Introduction

Relapse following allogeneic hematopoietic stem cell transplantation (HSCT) has remained unchanged over the past 3 decades, as reviewed in the second workshop convened by the National Institutes of Health (NIH).^2,3^

## EUROPEAN RESEARCH CONTRIBUTIONS AND PROPOSED RESEARCH FOR THE ROADMAP

### Disease phase

The strongest predictor of relapse is phase of the disease. Patients not in hematologic remission at the time of transplantation, or patients with minimal residual disease (MRD),^4,5^ have a high risk of relapse post-transplant. Thus, the first measure is to reduce tumor burden pretransplant.

### Prophylaxis pretransplant

A molecular remission pretransplant can be achieved with targeted therapy in acute lymphoid leukemia (ALL), using bispecific antibodies such as blinatumomab^6^ or chimeric antigen receptor T cells (CART)^7^: the role of allogeneic HSCT following CART CD19 therapy should be explored, to maintain the achievement of MRD negativity.

In patients with Flt3-positive acute myeloid leukemia (AML), midostaurin has shown encouraging results when used in induction.^8^ Several Flt3 inhibitors are now available and should be tested before allogeneic HSCT to induce MRD negativity. For patients with Flt3-negative AML, CART cell therapy needs to be expanded: a phase 1 trial with CART CD44v6 cells has been opened (ClinicalTrials.gov Identifier: NCT04097301). Further support needs to be given to the control of MRD in AML before HSCT.

The intensity of the conditioning regimen is another major player and should be further explored. In children with ALL, conventional total body irradiation (TBI) has been shown to be superior to chemotherapy (NCT01423747). The problem is the use of TBI in adults, and total marrow irradiation should be explored. In AML, thiotepa, busulfan, and fludarabine may reduce post-transplant relapse as compared with busulfan fludarabine.^9^ A prospective randomized trial has been designed to confirm this finding (NCT04217278).

Whether patients with myelodysplastic syndrome should be treated with azacytidine before an allogeneic HSCT remains uncertain: a randomized trial comparing upfront HSCT to azacytidine followed by HSCT has been activated (NCT04184505).

### Prophylaxis post-transplant

The role of post-HSCT prophylactic tyrosine kinase inhibitors for Philadelphia-positive ALL is still being discussed^10^ and needs to be assessed in prospective trials: a previous small, randomized study has shown improved leukemia control with prophylaxis as compared with MRD based, pre-emptive therapy.^11^ For patients with Flt3-positive AML, 2 recent randomized studies have shown significantly improved survival with post-HSCT prophylactic sorafenib,^12,13^ although this drug has currently no European Medicines Agency (EMA) approval in AML. Other Flt3 inhibitors such as gilteritinib need to be tested post-HSCT. A randomized trial with azacytidine to prevent leukemia relapse in AML has recently failed.^14^

Selection of optimal donor for leukemia control is an additional relevant issue, which is being addressed by 3 prospective randomized trials (NCT04232241, NCT04067180, and NCT03655145). These studies are designed to enroll almost 1000 patients, and we should then know whether a family haploidentical or an unrelated donor is best, for patients with acute leukemia.

The use of cellular therapy as prophylaxis should be considered: natural killer (NK) cells have shown to possess antitumor activity and may be given without the risk of graft versus host disease (GvHD).^15^ However, there is no proof that prophylactic infusions of NK cells will reduce leukemia relapse in acute leukemia. Prospective randomized trials testing the role of NK cell infusions in AML patients with active disease at transplant should be pursued (NCT 04166929).

### Treatment of hematologic relapse

The outcome of patients following a hematologic relapse will depend on the pace of the underlying disease. Patients with acute leukemia, relapsing within 6 months are at very high risk of early death, whereas delayed relapses can be successfully treated in a proportion of cases with a second transplant or chemotherapy and DLI.^2^ The use of bispecific antibodies and CART cells are expanding the therapeutic options for relapsed ALL patients and should be tested further.^16,17^ New treatment modalities such as the combination of azacytidine and venetoclax, for AML, should also be prospectively explored.

## ANTICIPATED IMPACT OF THE RESEARCH

Clinical trials need to be designed addressing (1) pre-transplant consolidation for MRD eradication and (2) the impact of conditioning regimen intensity on MRD clearance post HSCT. The selection of optimal donors (3) and conditioning regimens (4) should also be assessed prospectively. Post-transplant prophylaxis with drugs, cellular therapy, as well as monitoring of MRD must be improved, particularly in patients with acute leukemia, in whom time is crucial.

## THE HSC NICHE: REGULATION OF HSC FUNCTION AND SIGNIFICANCE FOR HSC EXPANSION

### Introduction

Hematopoiesis is critically regulated by nonhematopoietic cells that support the production of blood and immune cells according to the organismal demands. Mesenchymal stromal cells (MSCs) are key components of the hematopoietic microenvironment. Recent technological developments (most notably single-cell studies) have made it possible to describe different MSC subpopulations, several of which seem to show organ-specific properties and gene expression profiles. However, functional studies will be critical in the near future for fulfilling the perceived potential of stromal cells in immune modulation and tissue regeneration.

## EUROPEAN RESEARCH IN THE LAST 5 YEARS AND PROPOSED RESEARCH FOR ROADMAP

### Understanding the specific functions of MSC subsets

HSCs and their microenvironment represent probably the best-characterized hierarchical stem cell system in vertebrates. They have paved the path to understanding how cell lineages are organized, their fate determined, and how they contribute to function in other organs. Recent European research has revealed the cellular and spatial organization of different bone marrow MSC subsets.^18^ Computational methods have been developed to infer candidate gene networks in HSC-supporting MSCs^19^ and potential ligand–receptor interactions.^20^ Future studies will be essential to reveal the specific functions and potential of these MSC subpopulations.

### Understanding the Role of MSCs in Hematological Malignancies

Recent European research has demonstrated a key role for MSCs in the initiation, progression, and drug resistance of a variety of hematological neoplasms through paracrine or cell-contact–dependent mechanisms.^21^ Extracellular vesicles from lymphoma B cells render MSCs protumorigenic.^22^ Notch2 signaling on MSCs triggers Wnt-dependent survival of chronic lymphocytic leukemia.^23^ Particularly, Wnt5A from MSCs is required for the engraftment of normal and leukemic HSCs.^24^ MSCs remodel the extracellular matrix to support the progression of B-cell ALL,^25^ support the growth of myeloproliferative neoplasms^26^ or differentiate into myofibroblasts in myelofibrosis.^27^ Finally, mitochondrial transfer from MSCs protects ALL and AML cells from oxidative stress induced by chemotherapy.^28,29^ Therefore, elucidating and targeting niche-dependent mechanisms of tumorigenesis and resistance will be key to eradicate cancer.

### MSCs in Hematopoietic Regeneration

European research over the past 5 years has shown that MSCs enhance HSC mobilization for subsequent apheresis from peripheral blood and transplantation (HSCT).^30^ Targeting MSCs could help mitigate the damage to normal hematopoiesis by aging, AML, infection, or iron overload in beta-thalassemia.^31–34^ Additionally, MSC coinfusion could increase the efficiency of HSCT^35^ and minimize the risks of graft failure in gene therapy applications associated with low conditioning regimens and infusion of limited numbers of gene-edited HSCs.^36^

### MSCs in Tissue Regeneration, Immune Modulation, and Systemic Disease

MSCs have a profound impact on different immune cells, having therapeutic benefits in sepsis, autoimmune disorders or graft-versus-host disease (GVHD), and may influence the outcome of immunotherapies. Additionally, research on MSCs could influence the outcome and treatment of steadily increasing systemic conditions, such as diabetes, obesity, and aging.

## ANTICIPATED IMPACT OF THE RESEARCH

Increasing research in the hematopoietic microenvironment and particularly MSCs will, scientifically, feed into other stem cell systems and, clinically, provide new ways to modulate and treat hematopoietic diseases, immune responses, and regenerative processes. As a result of this productive research, Europe continues to be the world region with profound understanding of MSC contributions to controlling the hematopoietic niche and the composition of immune cell populations of each individual in health and disease.

## HUMAN PLURIPOTENT STEM CELLS: SOURCE OF (GENETICALLY REPAIRED) HSCS AND HEMATOPOIETIC PROGENITOR CELLS

### Introduction

**Figure 1.**
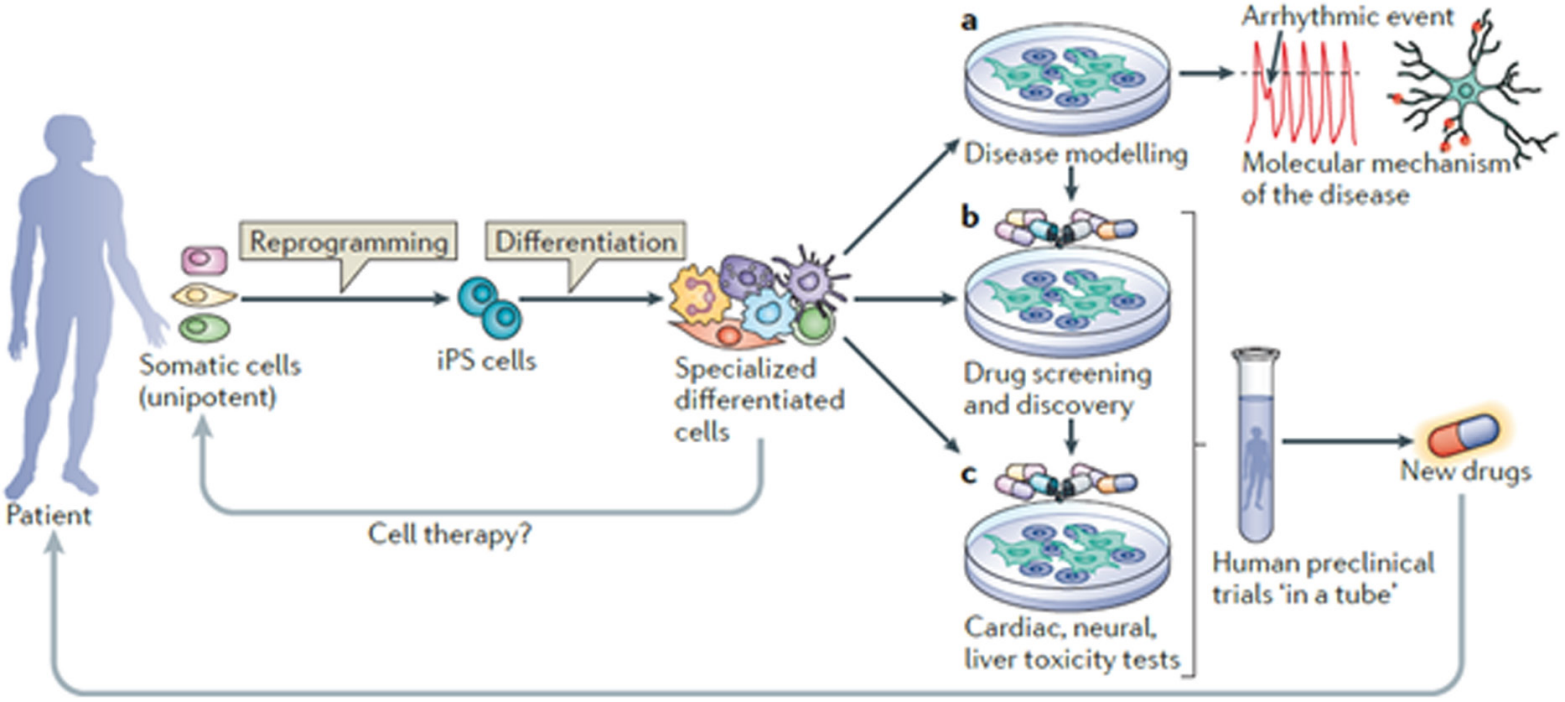
**Induced pluripotent stem cells: the new patient?** ^39^ Permission for reuse obtained from Nature/Springer.

The present availability of human pluripotent stem cells (hPSCs: see Figure 1) raises promise for a universal resource for cell based therapies in regenerative medicine. Rapid progress has been made in generating hPSCs amenable for clinical applications, culminating in reprogramming of adult somatic cells to autologous hPSCs that can be indefinitely expanded in vitro. However, a major challenge remains how to differentiate hPSCs to specific lineages efficiently and how to select for cells that will function normally upon transplantation in adults. Another major challenge, which until now has not been solved, is how to generate true HSCs that can be transplanted, engraft and maintain all (blood) stem cell properties. The hematopoietic lineage has been particularly refractory compared with other cell types and derivation of adult-type HSCs has not yet been possible even though various other blood lineages can be obtained.^37^ If HSCs could be generated from a patient’s own hPSCs, genetic engineering could easily be used to correct genetic defects before differentiation into transplantable HSCs, which would overcome some caveats of conventional HSCT therapies.

Skin fibroblasts were the first human somatic cells to be reprogrammed into hiPSC but blood cells are increasingly used because of the availability of banked (cord and peripheral) blood samples covering a range of disease, age, gender, and ethnic backgrounds. Complementing hiPSC generation are attempts at “direct reprogramming” using sets of lineage specific transcription factors that act as master regulators and convert cells to a new differentiation state without intermediate pluripotency. Generating hiPSC from mature B cells requires silencing of lineage-specific factors such as PAX5, so that direct reprogramming into blood cells where this would not be necessary, may therefore be quite feasible.^38^

The first steps in directed differentiation of hPSCs are guided by morphogens (eg, Wnt, transforming growth factor [TGF]-beta, activin, and bone morphogenetic protein [BMP]) which are important in development (Figure 1). hPSCs progress through a primitive-streak like stage before forming the 3 germ layers, endoderm, ectoderm, and mesoderm, which is the layer giving rise to (hemogenic) endothelial cells and blood. While early embryonic stages are quite faithfully mimicked in culture, patterning of the mesoderm requires anatomical structure of the embryo, embryonic cell-to-cell interactions, expression of patterning genes (*Cdx/Hox*), certain cell nonautonomous effects and exposure to physical stimuli (flow) that occur at specific stages of development. hPSC derivatives thus remain immature and, to date, all hPSC-derived HSCs have been deficient in their developmental potential, ability to self-renew and to engraft upon transplantation in mice, even though animal studies have shown that this is possible in the context of a developing embryo (where PSCs do have the innate ability to differentiate into fully functional, definitive HSCs). Normal HSC development during embryogenesis occurs in several distinct temporal/spatial waves, each characterized by its own set of hematopoietic progenitor cells (HPCs). Only those produced from the latter, or “definitive,” wave give rise to mature, functional HSCs but during in vitro differentiation, it is believed that the HSC-like cells produced are from the more primitive waves. These give rise to HSC-like cells biased toward myeloid lineages at the expense of lymphoid potential. These are unable to self-renew in culture and lack long-term engraftment capacity. Key to overcoming this HSC bottleneck will be understanding: (1) the ontogeny of human HSCs, (2) how they progress at the molecular level to become mature, adult HSCs, and (3) intrinsic and extrinsic factors that govern HSC behavior and function.

## EUROPEAN RESEARCH IN THE LAST 5 YEARS

Several prominent European hematologists are using genomic approaches (gene expression and microRNA profiles, transcription factor, and histone ChIP data) to examine the molecular mechanisms that regulate cell fate decisions (self-renewal versus differentiation, quiescence versus proliferation, and apoptosis) by HSCs or HPCs and their more differentiated progeny. This focus on genetic modification of stem cells, especially using gamma-retroviral and lentiviral vectors, has brought Europe in the global forefront of genetically modified stem cell therapy for treatment of various types of severe combined immunodeficiency (SCID). This has been in part through EU FP7 funded projects that included PERSIST, EuroStemCell, and NOVEXPAND, among others. This momentum is poised to combine HSC expansion with gene editing approaches that will move the field forwards toward innovative therapies in the coming decade. Several European consortia are currently applying for funding in the Horizon Europe scheme that has started 2021.

## PROPOSED RESEARCH FOR ROADMAP

An exciting new development in the gene therapy field is the use of designer nucleases, most recently CRISPR/Cas9 based, that are proving exceptionally efficient in engineering the genome.^40^ Recent study has established the potential for site specific gene editing to repair disease mutations in the human genome by targeting the integration of a corrective cDNA into the IL2RG locus of HSCs from a SCID-X1 patient.^41^ For homologous recombination-based gene editing approaches to be successful, HSC need to proliferate and template DNA harboring the corrective sequences needs to be efficiently introduced into the target cells. This can be readily achieved in hiPSC so that these cells are prime targets for gene editing techniques. However, as gene editing can also be done in primary HSCs, it is crucial to find conditions that allow HSC to proliferate without loss of phenotype. Therefore, HSC expansion protocols (see previous subsection, “European research in the last 5 years”) are directly relevant for gene repair described here. Niche signals, expansion, directed differentiation toward induced-HSCs (iHSCs), and genetic modification of HSCs and their progeny are related research topics that need an integrated approach for optimal exploitation in the clinical context.

Ex vivo generation of HSCs is currently difficult and requires ectopic expression of a number of transcription factors including *ERG*, *HOXA5*, *HOXA9*, *HOXA10*, *LCOR*, *RUNX1*, and *SPI*.^42^ However, the overexpression of such factors, in particular HOX genes, is associated with acute leukemogenesis. This indicates that more research is needed, not only for efficacy but also safety of hiPSC-derived iHSC. Careful single cell RNAseq, ATAC-seq and other -omics approaches comparing bona fide HSCs with iHSC may help in defining the relevant conditions to generate transplantable iHSC. Finally, in all of these approaches, residual undifferentiated hiPSC may also present a risk for clinical application of these cells.

## IMPACT OF PROPOSED RESEARCH

A major bottleneck to curing genetic diseases originating from mutations in HSCs is that even though efficient methods for gene targeting are now available, HSCs from neither adult nor hPSC sources can be expanded sufficiently for this to become routine practice. The combined projects in this section seek to address this, bringing safe gene therapy to the clinic via HSCs. New inroads into the generation of iHSC are needed to realize the true potential of these cells for clinical use in blood-mediated diseases.

**Summary box: Main research & policy priorities**

- Reduction of leukemia relapse remains a priority for future multimodal approach, including optimization of conditioning regimens and targeted drug therapy.
- An immunologic control of leukemia with cellular and/or antibody-based therapy needs to be expanded.
- Expansion of HSCs from either adult or hPSC sources in culture: even though efficient methods for gene targeting are now available, clinical use of these cells will require scaled cell production for the approach to become routine practice.
- More insight into the generation of iHSC (either by direct reprogramming or from hiPSCs) is needed to realize the full potential of these cells for clinical use in blood-mediated diseases.
- iHSC need to be engrafting upon transplantation, maintain self-renewal and multilineage differentiation.
- Costs of hPSC generation under good manufacturing practice (GMP) conditions need to be significantly reduced.
- Regulator engagement is essential early in the clinical development phase to guide products efficiently to the clinic.

## DISCLOSURES

WF is a chairman DSMB in the Glycostem Therapeutics and advisor for Starfish Innovations. DK receives research funding from Merck. CM received research funding from Sartorius. AB receives speaker’s fees from SANOFI, MSD, Therakos, Novartis, JAZZ, ADIENNE. All the other authors have no conflicts of interest to disclose.
